# Genome of the marine alphaproteobacterium *Hoeflea phototrophica* type strain (DFL-43^T^)

**DOI:** 10.4056/sigs.3486982

**Published:** 2013-02-25

**Authors:** Anne Fiebig, Silke Pradella, Jörn Petersen, Victoria Michael, Orsola Päuker, Manfred Rohde, Markus Göker, Hans-Peter Klenk, Irene Wagner-Döbler

**Affiliations:** 1 Leibniz Institute DSMZ – German Collection of Microorganisms and Cell Cultures, Braunschweig, Germany; 2 HZI – Helmholtz Center for Infection Research, Braunschweig, Germany

**Keywords:** aerobic, rod-shaped, motile, photoheterotroph, Phenotype MicroArray, bacteriochlorophyll *a*, symbiosis, dinoflagellates, *Prorocentrum lima*, *Phyllobacteriaceae*

## Abstract

*Hoeflea phototrophica* Biebl *et al*. 2006 is a member of the family *Phyllobacteriaceae* in the order *Rhizobiales*, which is thus far only partially characterized at the genome level. This marine bacterium contains the photosynthesis reaction-center genes *pufL* and *pufM* and is of interest because it lives in close association with toxic dinoflagellates such as *Prorocentrum lima*. The 4,467,792 bp genome (permanent draft sequence) with its 4,296 protein-coding and 69 RNA genes is a part of the **M**arine **M**icrobial **I**nitiative.

## Introduction

Strain DFL-43^T^ (= DSM 17068 = NCIMB 14078) is the type strain of *Hoeflea phototrophica*, a marine member of the *Phyllobacteriaceae* (*Rhizobiales, Alphaproteobacteria*) [1]. The genus, which was named in honor of the German microbiologist Manfred Höfle [2], contains four species, with *H. marina* as type species [2]; the name of a fifth member of the genus, '*Hoeflea siderophila',* is until now only effectively published [3]. *H. phototrophica* DFL-43^T^ and strain DFL-44 were found in the course of a screening program for marine bacteria containing the photosynthesis reaction-center genes *pufL* and *pufM* [4]. The species epithet '*phototrophica*' refers to the likely ability of *H. phototrophica* strains to use light as an additional energy source [1]. Strain DFL-43^T^ was isolated from single cells of a culture of the toxic dinoflagellate *Prorocentrum lima* maintained at the Biological Research Institute of Helgoland, Germany [1]. Here we present a summary classification and a set of features for *H. phototrophica* DFL-43^T^ including so far undiscovered aspects of its phenotype, together with the description of the complete genomic sequencing and annotation.

This work is part of the Marine Microbial Initiative (MMI) which enabled the J. Craig Venter Institute (JCVI) to sequence the genomes of approximately 165 marine microbes with funding from the Gordon and Betty Moore Foundation. These microbes were contributed by collaborators worldwide, and represent an array of physiological diversity, including carbon fixers, photoautotrophs, photoheterotrophs, nitrifiers, and methanotrophs. The MMI was designed to complement other ongoing research at JCVI and elsewhere to characterize the microbial biodiversity of marine and terrestrial environments through metagenomic profiling of environmental samples.

## Classification and features

### 16S rRNA analysis

A representative genomic 16S rRNA sequence of *H. phototrophica* DFL-43^T^ was compared using NCBI BLAST [5,6] under default settings (e.g., considering only the high-scoring segment pairs (HSPs) from the best 250 hits) with the most recent release of the Greengenes database [7] and the relative frequencies of taxa and keywords (reduced to their stem [8]) were determined, weighted by BLAST scores. The most frequently occurring genera were *Rhizobium* (53.7%), *Sinorhizobium* (24.0%), *Hoeflea* (4.5%), *Bartonella* (4.5%) and *Ahrensia* (3.7%) (132 hits in total). Regarding the two hits to sequences from members of the species, both, the average identity within HSPs and the average coverage by HSPs were 100.0%. Regarding the single hit to sequences from other members of the genus, the average identity within HSPs was 98.2%, whereas the average coverage by HSPs was 100.0%. Among all other species, the one yielding the highest score was *H. marina* (AY598817), which corresponded to an identity of 98.2% and an HSP coverage of 100.0%. (Note that the Greengenes database uses the INSDC (= EMBL/NCBI/DDBJ) annotation, which is not an authoritative source for nomenclature or classification.) The highest-scoring environmental sequence was AY922224 (Greengenes short name 'whalefall clone 131720'), which showed an identity of 98.1% and an HSP coverage of 97.5%. The most frequently occurring keywords within the labels of all environmental samples which yielded hits were 'bee' (3.1%), 'singl' (3.0%), 'abdomen, bumbl, distinct, honei, microbiota, simpl' (2.9%), 'microbi' (2.8%) and 'structur' (1.8%) (118 hits in total). Environmental samples which yielded hits of a higher score than the highest scoring species were not found, indicating that *H. phototrophica* is rarely found in environmental samples.

**Figure 1:**
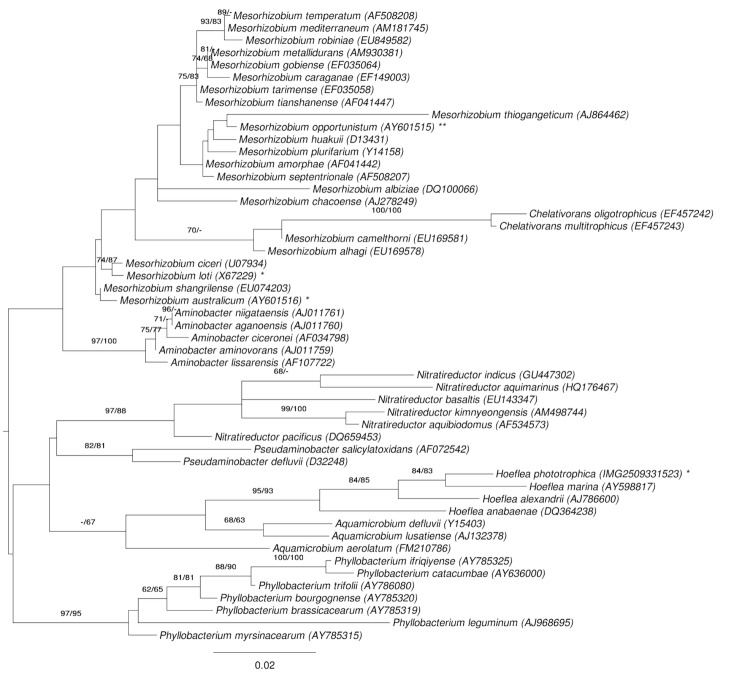
Phylogenetic tree highlighting the position of *H. phototrophica* relative to the type strains of the other species within the family *Phyllobacteriaceae*. The tree was inferred from 1,362 aligned characters [9,10] of the 16S rRNA gene sequence under the maximum likelihood (ML) criterion [11]. Rooting was done initially using the midpoint method [12] and then checked for its agreement with the current classification (Table 1). The branches are scaled in terms of the expected number of substitutions per site. Numbers adjacent to the branches are support values from 1,000 ML bootstrap replicates [13] (left) and from 1,000 maximum-parsimony bootstrap replicates [14] (right) if larger than 60%. Lineages with type strain genome sequencing projects registered in GOLD [15] are labeled with one asterisk, those also listed as 'Complete and Published' (CP002279 for *Mesorhizobium opportunistum*) with two asterisks.

Figure 1 shows the phylogenetic neighborhood of *H. phototrophica* in a 16S rRNA based tree. The sequences of the two identical 16S rRNA gene copies in the genome differ by one nucleotide from the previously published 16S rRNA sequence (AJ582088)

**Table 1:** Classification and general features of *H. phototrophica* DFL-43^T^ according to the MIGS recommendations [16].

MIGS ID	Property	Term	Evidence code
	Current classification	Domain *Bacteria*	TAS [17]
		Phylum *Proteobacteria*	TAS [18]
		Class *Alphaproteobacteria*	TAS [19,20]
		Order *Rhizobiales*	TAS [20,21]
		Family *Phyllobacteriaceae*	TAS [20,22]
		Genus *Hoeflea*	TAS [2]
		Species *Hoeflea phototrophica*	TAS [1]
MIGS-7	Subspecific genetic lineage (strain)	DFL-43^T^	TAS [1]
MIGS-12	Reference for biomaterial	Biebl *et al.* 2006	TAS [1]
	Gram stain	Gram-negative	TAS [1]
	Cell shape	rod-shaped	TAS [1]
	Motility	motile	TAS [1]
	Sporulation	not reported	
	Temperature range	mesophile, 25-33°C	TAS [1]
	Optimum temperature	31°C	TAS [1]
	Salinity	0.5–7.0 % NaCl	TAS [1]
MIGS-22	Relationship to oxygen	aerobe	TAS [1]
	Carbon source	acetate, malate	TAS [1]
	Energy metabolism	photoheterotroph	TAS [1]
MIGS-6	Habitat	marine	TAS [1]
MIGS-6.2	pH	6–9.0	TAS [1]
MIGS-15	Biotic relationship	host-associated	TAS [1]
MIGS-14	Known pathogenicity	none	TAS [1]
MIGS-16	Specific host	*Prorocentrum lima* ME130	TAS [1]
MIGS-18	Health status of Host	not reported	
	Biosafety level	1	TAS [23]
MIGS-19	Trophic level	not reported	
MIGS-23.1	Isolation	from a culture of *Prorocentrum lima* ME130	TAS [1]
MIGS-4	Geographic location	not reported	
MIGS-5	Time of sample collection	April 1, 2002	TAS [1]
MIGS-4.1	Latitude	54.133	TAS [1]
MIGS-4.2	Longitude	7.867	TAS [1]
MIGS-4.3	Depth	not reported	
MIGS-4.4	Altitude	not reported	

Evidence codes TAS: Traceable Author Statement (i.e., a direct report exists in the literature); NAS: Non-traceable Author Statement (i.e., not directly observed for the living, isolated sample, but based on a generally accepted property for the species, or anecdotal evidence). Evidence codes are from the Gene Ontology project [24].

### Morphology and physiology

**Figure 2:**
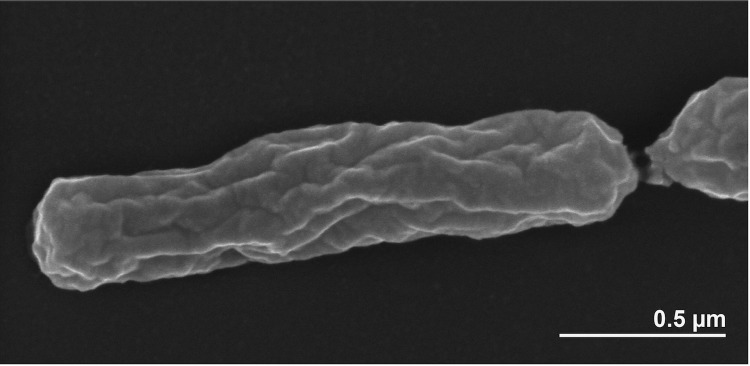
Scanning electron micrograph of *H. phototrophica* DFL-43^T^

Cells of *H. phototrophica* are small rods of 0.3–0.5 *μ*m in width and 0.7–2.0 μm length [1] (Figure 2) and motile by means of single, polar flagellum [1] (not visible in Figure 2). Depending on the availability of light, colonies are opaque to beige (grown in the dark) on marine agar 2216 [1]. The cultures are strictly aerobic and prefer microaerobic conditions. Good growth was detectable within a range of 25-33°C (1/5 limited growth rate below this value), concentration of sea salt from 0.5-7.0% and pH values from 6.0-9.0 [1]. Acetate and malate were accepted as carbon sources, whereas ethanol and methanol were not used for growth [1]. No hydrolysis of gelatin, starch, alginate or Tween 8 was observed [1].

The utilization of carbon compounds by *H. phototrophica* DFL-43^T^ was also determined for this study using PM01 microplates in an OmniLog phenotyping device (BIOLOG Inc., Hayward, CA, USA). The microplates were inoculated at 28°C with a cell suspension at a cell density of 85% Turbidity and dye D. Further additives were artificial sea salts, vitamins, trace elements and NaHCO_3_. The exported measurement data were further analyzed with the opm package for R [25], using its functionality for statistically estimating parameters from the respiration curves such as the maximum height, and automatically translating these values into negative, ambiguous, and positive reactions. The strain was studied in two independent biological replicates, and reactions with a different behavior between the two repetitions were regarded as ambiguous and are not listed below.

*H. phototrophica* DFL-43^T^ was positive for D,L-malic acid, D-cellobiose, D-fructose, D-galactonic acid-γ-lactone, D-galactose, D-galacturonic acid, D-gluconic acid, D-glucuronic acid, D-malic acid, D-mannitol, D-melibiose, D-sorbitol, D-trehalose, D-xylose, L-alanine, L-arabinose, L-glutamic acid, L-glutamine, L-lactic acid, L-lyxose, L-malic acid, L-proline, L-serine, acetic acid, adonitol, α-D-glucose, α-keto-glutaric acid, α-methyl-D-galactoside, β-methyl-D-glucoside, bromo-succinic acid, citric acid, ethanolamine, fumaric acid, m-inositol, maltose, maltotriose, mono-methyl succinate, propionic acid, pyruvic acid, succinic acid, sucrose and uridine. The strain was negative for 1,2-propanediol, 2'-deoxy-adenosine, D,L-α-glycerol-phosphate, D-alanine, D-aspartic acid, D-fructose-6-phosphate, D-glucosaminic acid, D-glucose-1-phosphate, D-glucose-6-phosphate, D-mannose, D-psicose, D-serine, D-threonine, L-alanyl-glycine, L-aspartic acid, L-fucose, L-galactonic acid-γ-lactone, L-rhamnose, L-threonine, N-acetyl-D-glucosamine, N-acetyl-β-D-mannosamine, acetoacetic acid, adenosine, α-D-lactose, α-hydroxy-butyric acid, α-hydroxy-glutaric acid-γ-lactone, α-keto-butyric acid, β-phenylethylamine, dulcitol, glycolic acid, glycyl-L-aspartic acid, glyoxylic acid, inosine, m-hydroxy-phenylacetic acid, m-tartaric acid, mucic acid, thymidine, tricarballylic acid, tween 40, tween 80 and tyramine.

### Chemotaxonomy

Phosphatidylglycerol, phosphatidylethanolamine and phosphatidylmonomethylethanolamine were the predominant polar lipids of the membrane. The most frequent cellular fatty acids in strain DFL-43^T^ are the mono-unsaturated straight chain acids C_18:1 ω7_ (62.8%) and its methylated form C_18:1 ω7 11Me_ (21%), followed by C_16:0_ (6.3%) and C_19:1_ (3.4%) [1]. The absorption spectrum of an acetone/methanol extract showed the presence of bacteriochlorophyll a and an additional carotenoid (possibly spheroidenone) in small amounts [1]. Further experiments indicated that the pigment production depends on the concentration of sea salts in the medium [1].

## Genome sequencing and annotation

### Genome project history

The genome was sequenced within the MMI supported by the Gordon and Betty Moore Foundation. Initial Sequencing was performed by the J. Craig Venter Institute, JCVI (Rockville, MD, USA), and a high-quality draft sequence was deposited at INSDC. The number of scaffolds and contigs was reduced and the assembly improved by a subsequent round of manual gap closure at HZI/DSMZ. A summary of the project information is shown in Table 2.

**Table 2:** Genome sequencing project information

**MIGS ID**	**Property**	**Term**
MIGS-31	Finishing quality	High quality draft
MIGS-28	Libraries used	Two genomic libraries: 40 kb fosmid library and 3 kb pUC18 plasmid library
MIGS-29	Sequencing platform	ABI3730
MIGS-31.2	Sequencing coverage	10.3 × Sanger
MIGS-30	Assemblers	Consed 20.0
MIGS-31.3	Contig count	5
MIGS-32	Gene calling method	Prodigal 2.0, Infernal 1.0.2
	INSDC ID	Final ID pending; previous version ABIA00000000
	Genbank Date of Release	final version not yet available
	GOLD ID	Gi01415
	NCBI project ID	19311
	Database: IMG	2509276008
MIGS-13	Source Material Identifier	DSM 17068
	Project relevance	Environmental, Marine Microbial Initiative

### Growth conditions and DNA extractions

Cells of strain DFL-43^T^ were grown for two to three days on a LB & sea-salt agar plate, containing (l^-1^) 10 g tryptone, 5 g yeast extract, 10 g NaCl, 17 g sea salt (Sigma-Aldrich S9883) and 15 g agar. A single colony was used to inoculate LB & sea-salt liquid medium and the culture was incubated at 28°C on a shaking platform. The genomic DNA was isolated using the Qiagen Genomic 500 DNA Kit (Qiagen 10262) as indicated by the manufacturer. DNA quality and quantity were in accordance with the instructions of the genome sequencing center. DNA is available through the DNA Bank Network [26].

### Genome sequencing and assembly

The genome was sequenced with the Sanger technology using a combination of two libraries. All general aspects of library construction and sequencing performed at the JCVI can be found on the JCVI website. Base calling of the sequences were performed with the phredPhrap script using default settings. The reads were assembled and assemblies analyzed using the phred/phrap/consed pipeline [27]. The last gaps were closed by adding new reads produced by recombinant PCR and PCR primer walks. In total 21 Sanger reads were required for gap closure and improvement of low quality regions. The final consensus sequence was built from 46,086 Sanger reads (10.3 × coverage).

### Genome annotation

Gene prediction was carried out using GeneMark as part of the genome annotation pipeline in the Integrated Microbial Genomes Expert Review (IMG-ER) system [28]. To identify coding genes, Prodigal [29] was used, while ribosomal RNA genes within the genome were identified using RNAmmer [30]. Other non-coding genes were predicted using Infernal [31]. Manual functional annotation was performed within the IMG platform [28] and the Artemis Genome Browser [32].

## Genome properties

**Figure 3:**
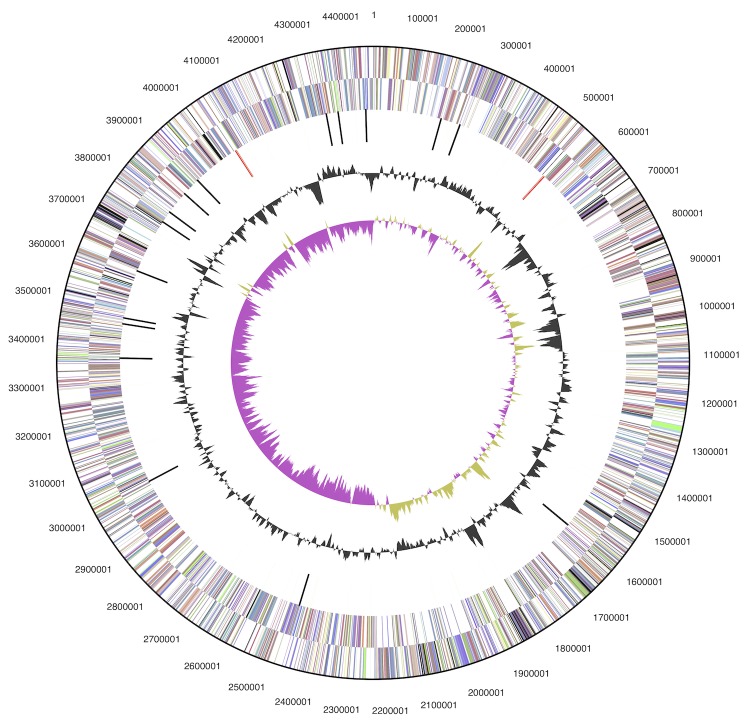
Graphical map of the chromosome. From outside to the centerp: Genes on forward strand (color by COG categories), RNA genes (tRNAs green, rRNAs red, other RNAs black), GC content, GC skew.

The draft genome consists of one circular scaffold with a total length of 4,467,822 bp containing five large contigs with a total length of 4,467,792 bp and a G+C content of 59.8%. Contig lengths vary from 133,683 bp to 2,215,172 bp (Figure 3); genome statistics are provided in Table 3. Of the 4,296 genes predicted, 4,227 were protein-coding genes, and 69 RNAs; pseudogenes were not identified. The majority of the protein-coding genes (83.1%) were assigned a putative function while the remaining ones were annotated as hypothetical proteins. The distribution of genes into COGs functional categories is presented in Table 4.

**Table 3:** Genome Statistics

**Attribute**	**Value**	**% of Total**
Genome size (bp)	4,467,832	100.00
DNA coding region (bp)	4,006,040	89.66
DNA G+C content (bp)	2,671,973	59.81
Number of replicons	1	
Extrachromosomal elements	0	
Total genes	4,296	100.00
RNA genes	69	1.61
rRNA operons	2	
tRNA genes	47	1.09
Protein-coding genes	4,227	98.39
Pseudo genes	0	
Genes with function prediction	3,574	83.19
Genes in paralog clusters	1,423	33.12
Genes assigned to COGs	3,525	82.05
Genes assigned Pfam domains	3,580	83.33
Genes with signal peptides	927	21.58
Genes with transmembrane helices	994	24.57
CRISPR repeats	0	

**Table 4:** Number of genes associated with the general COG functional categories

**Code**	**Value**	**%age**	**Description**
**J**	178	4.58	Translation, ribosomal structure and biogenesis
**A**	0	0.00	RNA processing and modification
**K**	274	7.05	Transcription
**L**	162	4.17	Replication, recombination and repair
**B**	2	0.05	Chromatin structure and dynamics
**D**	27	0.69	Cell cycle control, cell division, chromosome partitioning
**Y**	-	-	Nuclear structure
**V**	39	1.00	Defense mechanisms
**T**	175	4.50	Signal transduction mechanisms
**M**	205	5.27	Cell wall/membrane/envelope biogenesis
**N**	60	1.54	Cell motility
**Z**	0	0.00	Cytoskeleton
**W**	-	-	Extracellular structures
**U**	66	1.70	Intracellular trafficking, secretion, and vesicular transport
**O**	135	3.47	Posttranslational modification, protein turnover, chaperones
**C**	226	5.81	Energy production and conversion
**G**	325	8.36	Carbohydrate transport and metabolism
**E**	405	10.41	Amino acid transport and metabolism
**F**	80	2.06	Nucleotide transport and metabolism
**H**	157	4.04	Coenzyme transport and metabolism
**I**	188	4.83	Lipid transport and metabolism
**P**	178	4.58	Inorganic ion transport and metabolism
**Q**	130	3.34	Secondary metabolites biosynthesis, transport and catabolism
**R**	524	13.47	General function prediction only
**S**	353	9.08	Function unknown
**-**	773	18.00	Not in COGs
